# Higher Rates of Hemolysis Are Not Associated with Albuminuria in Jamaicans with Sickle Cell Disease

**DOI:** 10.1371/journal.pone.0018863

**Published:** 2011-04-14

**Authors:** Monika R. Asnani, Raphael A. Fraser, Marvin E. Reid

**Affiliations:** Sickle Cell Unit, Tropical Medicine Research Institute, Mona Campus, University of the West Indies, Kingston, Jamaica; INSERM, France

## Abstract

**Background:**

Albuminuria is a marker of glomerular damage in Sickle Cell Disease (SCD). In this study, we sought to determine the possible predictors of albuminuria in the two more prevalent genotypes of SCD among the Jamaica Sickle Cell Cohort Study participants.

**Methods:**

An age-matched cohort of 122 patients with HbSS or HbSC genotypes had measurements of their morning urine albumin concentration, blood pressure, body mass index, haematology and certain biochemistry parameters done. Associations of albuminuria with possible predictors including hematological parameters, reticulocyte counts, aspartate aminotransferase (AST) and lactate dehydrogenase (LDH) levels were examined using multiple regression models.

**Results:**

A total of 122 participants were recruited (mean age 28.6 years ±2.5 years; 85 HbSS, 37 HbSC). 25.9% with HbSS and 10.8% with HbSC disease had microalbuminuria (urine albumin/creatinine ratio  =  30–300 mg/g of creatinine) whereas 16.5% of HbSS and 2.7% of HbSC disease had macroalbuminuria (urine albumin/creatinine ratio>300 mg/g of creatinine). Mean arterial pressure, hemoglobin levels, serum creatinine, reticulocyte counts and white blood cell counts were statistically significant predictors of albuminuria in HbSS, whereas white blood cell counts and serum creatinine predicted albuminuria in HbSC disease. Both markers of chronic hemolysis, i.e. AST and LDH levels, showed no associations with albuminuria in either genotype.

**Conclusions:**

Renal disease, as evidenced by excretion of increased amounts of albumin in urine due to a glomerulopathy, is a common end-organ complication in SCD. It is shown to be more severe in those with HbSS disease than in HbSC disease. Rising blood pressure, lower hemoglobin levels and higher white blood cell counts are hints to the clinician of impending renal disease, whereas higher rates of hemolysis do not appear to play a role in this complication of SCD.

## Introduction

Chronic renal failure is common in the Jamaican population with a prevalence of 327 per million persons [1]. Sickle cell disease (SCD) is the commonest hemoglobinopathy in Jamaica with an incidence of 1∶150 live births. Renal failure in turn is also common among subjects with SCD, and constitutes ∼1% of the cases of renal dysfunction in the island [1]. Renal failure has contributed to about 18% of death in Jamaican patients over 40 years of age with the homozygous SS (HbSS) disease [2]. As improved clinical management of patients reflects into longer life span, renal failure will play an increasing role in their morbidity and mortality. Once end-stage renal disease is diagnosed, survival thereafter is limited to about 4 years [3].

Proteinuria is known to be a frequent finding in SCD [4], and albuminuria may infact be an early marker of glomerular disease leading ultimately to renal failure [5]. In general, there is evidence suggesting that proteinuria itself may induce further nephrotoxicity [6], [7]. In SCD, major determinants of the prevalence of proteinuria are age and genotype. In general the prevalence of proteinuria increases with age and is higher in HbSS compared with heterozygous SC (HbSC) genotype [3], [8]. Microalbuminuria has been reported in 6.2% of pediatric patients with HbSS; but 10% in teenagers with HbSS [9]. In adults, the prevalence rises to 17–49% of those with HbSS [10], [11]. The clinical relevance and the pathogenic mechanisms underlying albuminuria in SCD are unclear but increased glomerular filtration rate (GFR) and renal plasma flows are noted in early years [12], [13]. It has been proposed that this hyper-filtration leads to gradual sclerosis of the glomerular capillaries and predisposes to renal insufficiency in these patients [12], [14]. Also the use of angiotensin converting enzyme (ACE) inhibitors, which reduces glomerular hyperfiltration, has been reported to be effective in reducing proteinuria in sickle cell disease [11], [15], [16]. Within this context, screening, assessment for possible renal damage and identification of modifiable risks factors could help improve outcome. In this study, we sought to determine the prevalence of albuminuria in patients with HbSS and HbSC from the Jamaica Sickle Cell Cohort Study (JSCCS), as well as to determine its predictors and in particular the association of albuminuria with increasing rates of hemolysis.

## Methods

A descriptive, observational, cross- sectional study was performed at the Sickle Cell Unit (SCU) of the Tropical Medicine Research Institute, The University of the West Indies from January - February 2006.

### Ethics Statement

The study was granted ethical approval by the University of the West Indies/University Hospital of the West Indies Ethical Committee. The study was designed and performed in adherence with the Declaration of Helsinki. Written, informed consent was obtained from all participants involved in the study.

### Participants

These patients have attended the SCU and have participated in the JSCCS since birth [17]. At the 2006 annual review, 122 patients with HbSS or HbSC were studied. Inclusion criteria included a mid stream urine test negative for infection, and none were presently on angiotensin converting enzyme inhibitor (ACE inhibitor) or angiotensin receptor blocker (ARB) therapy. All participants were clinically healthy at the time of the study.

### Measurements

The participants had measurements of their morning urine albumin concentration using an immunoturbidimetric assay on Alcyon™ 300 Falcor. Three measurements of sitting blood pressure and pulse rate were taken at 5-minute intervals using a Dynamap®. The averages of the three determinations were used in the study. Mean arterial pressure was calculated from the mean systolic and diastolic blood pressure values as [1/3(Systolic pressure) + 2/3(Diastolic pressure)].

Height was measured with a wall- mounted Staidiometer and weight was measured using a lever balance.

Blood samples were collected from each subject for various hematological and biochemical measurements and stored at −70°C until analysis was done.

### Statistical Analysis

Descriptive statistics e.g. means, standard deviations and frequency counts were used as appropriate. We used two sample t-tests to test for differences between means in continuous outcome measurements by genotype.

Random urine albumin values were left censored at <4.0 mg/L as this represented the lower limits of detection for the albumin assay. Fifty seven (47%) observations were censored and for them, we set the value of albumin concentration to 2.0 (4.0/2) mg/L. Albumin- creatinine ratios (ACR) were calculated using this value for albumin concentrations. Microalbuminuria was defined as ACR 30–300 mg/g of creatinine, and macroalbuminuria as >300 mg/g of creatinine. As the distribution of the ACR was right skewed, it was normalized using log transformation. To determine the predictors of albuminuria, a series of multiple linear regressions with log ACR as outcome were performed. The potential predictors included hematology, anthropometry, blood pressure, age, and gender. Predictors of albuminuria were selected using a manual procedure after adjusting for gender. Models were compared using the partial F statistic. As the distributions of hematological variables were different by genotype, regressions models incorporating these variables were performed separately by genotype. Significance was defined at an alpha level of 0.05.

Data was analyzed using Stata Software version 10.0 for Windows™ (StataCorp, College Station, TX).

## Results

A total of 122 participants were included in the study. Of these, 69.7% (50 males, 35 females) were of HbSS genotype and 30.3% (22 males, 15 females) were HbSC. The mean age of the sample was 28.6 +/− 2.5 years with a range from 24.1–32.5 years. There was no statistical significant difference in age between the two genotypes.

### General characteristics

There were statistically significant differences between all the potential predictor variables by genotype with the exception of age, height, systolic blood pressure and serum iron. HbSS patients had lower weight, BMI, diastolic blood pressure, mean arterial pressure, hemoglobin, lower sodium and serum creatinine than their HbSC counterparts. However, they had higher fetal hemoglobin, absolute reticulocyte counts, white blood cell counts, lactate dehydrogenase (LDH) and aspartate aminotransferase (AST) (the last two as a markers of hemolysis) (Table 1). Almost 29% of this group of HbSS patients had a lifetime history of having had chronic leg ulceration, i.e. a leg ulcer lasting 6 months or longer. Majority (56.5%) of our HbSS sample had the normal complement of α genes (αα/αα) whereas 40% had a single α gene deletion, i.e. heterozygous α-thallassemia (αα/α-). The data on leg ulcer prevalence and α gene status was not available for the HbSC sample.

**Table 1:** Summary of anthropometry, hematology, biochemistry and blood pressure characteristics by genotype.

	SS (n = 85)		SC (n = 37)			
	Mean	SD	Mean	SD	*P*-value	95% Confidence Interval
**Age (years)**	28.5	2.6	28.9	2.5	0.38	−0.6 to 1.4
**Height (cm)**	170.0	8.4	169.5	8.6	0.75	−3.8 to 2.8
**Weight (Kg)**	57.7	10.1	67.0	14.6	<0.001	4.7 to 13.8
**BMI (Kg/m^2^)**	20.0	3.0	23.2	4.4	<0.001	2.0 to 4.6
**Systolic BP (mm Hg)**	108.0	11.2	107.6	11.2	0.86	−4.8 to 4.0
**Diastolic BP (mm Hg)**	62.5	11.2	70.0	9.8	<0.001	3.3 to 11.7
**Mean arterial pressure (mmHg)**	77.8	10.4	82.5	9.3	0.02	0.78 to 8.7
**Fetal hemoglobin (%)**	4.7	3.5	2.0	2.3	<0.001	−4.0 to −1.5
**Hemoglobin (g/dL)**	8.2	1.4	12.3	1.5	<0.001	3.5 to 4.6
**Absolute Retics (10^6^/L)**	364.5	212.9	263.3	118.1	0.009	−176.1 to −26.3
**White blood cells (×10^9^/L)**	11.0	3.9	7.7	3.0	<0.001	−4.8 to −1.9
**Lactate dehydrogenase (U/L)**	404.0	143.6	225.6	63.8	<0.001	−227.2 to −130.0
**Serum creatinine (µmol/L)**	53.0	14.3	72.7	14.4	<0.001	14.2 to 25.4
**Sodium (mmol/L)**	132.4	10.1	136.5	5.9	0.02	0.56 to 7.8
**Serum Iron**	12.8	5.1	11.8	3.7	0.29	−2.9 to 0.9
**Aspartate Aminotransferase (U/L)**	39.2	17.8	22.0	6.9	<0.001	−23.8 to −10.6

### Urinary albumin excretion

**Figure 1:**
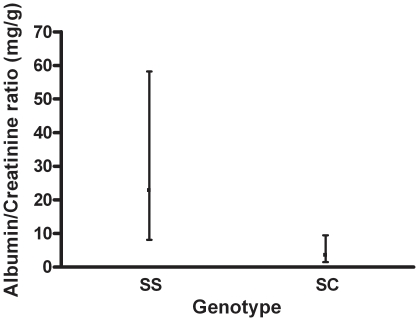
Albumin/creatinine ratios with SDs for SS and SC genotypes. Values are geometric means with 95% confidence intervals.

HbSS patients had significantly higher urinary albumin/creatinine ratios than those with HbSC disease (Figure 1). Microalbuminuria was found in 25.9% of patients with HbSS, as compared to 10.8% of those with HbSC disease, whereas macroalbuminuria was present in 16.5% of HbSS and 2.7% of HbSC patients. Within HbSS, those with a history of chronic leg ulceration had a significantly higher prevalence of albuminuria (40% albuminuria vs. 20% albuminuria, p value: 0.05), whereas there was no difference in albuminuria by α gene status (Table 2).

**Table 2:** Prevalence of Leg Ulceration and α-Thal trait vs. albuminuria status in SS genotype.

	Albuminuria Status		p-value
	Positive	Negative	
**Leg Ulceration, n(%)**YesNo	14 (40.0)21 (60.0)	10 (20.4)39 (79.6)	0.050
**α-Thal Status, n(%)** ** α -/α –** ** α -/αα** ** αα/αα**	1 (2.8)12 (33.3)23 (63.9)	2 (4.1)22 (44.9)25 (51.0)	0.496

### Factors associated with albuminuria

The distribution of the hematological variables by genotype was distinct, i.e. overlap was minimal. Hence separate multiple linear regression analyses were performed for each genotype to facilitate inclusion of these variables. For persons with HbSS, higher mean arterial pressure (p value <0.001), higher white blood cell counts (p value 0.003), lower hemoglobin (p value 0.03), lower absolute reticulocyte counts (p value 0.02) and lower serum creatinine (p value 0.04) were significant predictors of higher albumin excretions in the urine (Table 3). This model accounted for almost 33% of the variance observed in the outcome.

**Table 3:** Factors associated with albuminuria in persons with SS disease.

Variable	Coef.	P-value	95% confidence interval
**Male sex**	−0.24	0.57	−1.08 to 0.60
**Absolute Retics (10^6^/L)**	−0.002	0.018	−0.004 to −0.001
**White blood cells(×10^9^/L)**	0.15	0.003	0.05 to 0.25
**Serum creatinine (µmol/L)**	−0.033	0.038	−0.06 to −0.002
**Hemoglobin (g/dl)**	−0.33	0.027	−0.63 to −0.04
**Mean arterial pressure (mmHg)**	0.09	<0.001	0.05 to 0.13
**Constant**	−4.36	0.02	−8.09 to −0.63

**Adjusted R^2^: 33.1; Prob>F: <0.001.**

For persons with HbSC, higher white blood cell counts (p value 0.013) and higher serum creatinine levels (p value 0.02) predicted greater levels of albuminuria (Table 4). This model accounted for about 33.5% of the variance observed in the outcome. Gender, blood pressure and hemoglobin levels were not seen to be significant predictors in this genotype.

**Table 4:** Factors associated with albuminuria in persons with SC disease.

Variable	Coef.	P-value	95% confidence interval
**Male sex**	−0.55	0.28	−1.52 to 0.45
**White blood cells(×10^9^/L)**	0.22	0.013	0.05 to 0.38
**Serum creatinine (µmol/L)**	0.04	0.02	0.01 to 0.08
**Constant**	−3.18	0.01	−5.51 to −0.85

**Adjusted R^2^: 33.5; Prob>F: 0.001.**

## Discussion

In this study, albuminuria was present in 42.3% of HbSS and 13.5% of HbSC patients. This compares quite well to past studies [10], [11] in HbSS. Past studies of albuminuria in HbSC have been scant and have reported levels of 9% (1 out of 11 patients studied) [18]. The present study is especially important as it offers a view of renal dysfunction in a cohort of patients with SCD which has been actively followed up since birth. In fact, the same cohort of HbSS patients had albuminuria levels measured at 26% ten years before this study [19] indicating a worsening glomerulopathy. Hemoglobin is once again shown to be negatively associated with albuminuria in those with HbSS, as it was in the study 10 years ago, even though Guasch et al. have shown nil relation of hemoglobin with albuminuria. They did however show significantly lower levels of hemoglobin once their patients had overt renal insufficiency [20]. Undoubtedly, proteinuria, increasing blood pressures and worsening anemia have been shown to precede overt renal failure in persons with HbSS in past studies [3].

Mean arterial pressure is seen to be associated with increasing albuminuria in persons with HbSS. Higher blood pressures have been shown to be associated with increased mortality [21], and one mechanism may be through renal dysfunction. The study by Powars et al. [3] has shown that whereas higher blood pressures accompanied (and in fact preceded chronic renal failure) all those who had renal failure, none of their controls (those without renal failure) had high blood pressure. It is important therefore clinically to be alert to rising blood pressure values, even to levels that are normal in those without SCD, as they will usually signify impending renal insufficiency.

LDH is known to be a marker of increasing severity in SCD and there are strong epidemiological associations between its elevation and both endothelial activation and pulmonary hypertension [22]. Data suggest that high LDH values may predict early mortality. However, neither LDH nor AST levels (both of which indicate hemolysis) have shown any association with albuminuria for either genotype in this study, implying that mechanisms implicated in pulmonary hypertension and sickle nephropathy may be different. Guasch et al. [20] have also reported no association between LDH and albuminuria, whereas Taylor et al. [23] have reported no association of LDH with glomerular filtration rates. On the other hand Gurkan et al. [24] have reported a significant association of albuminuria with LDH and Maier-Redelsperger and colleagues [25] have found a composite variable, composed of LDH and reticulocytes, to be highly correlated to albuminuria.

We are further reporting that absolute reticulocyte counts are significant negative predictors of albuminuria in those with HbSS disease. This is contrary to the findings of Guasch et al. who reported no association of reticulocytes with albuminuria. They however conducted their analyses using percentage of reticulocytes which may not necessarily indicate reticulocytosis. Haymann et al. [26] have in their study demonstrated a positive relationship between glomerular filtration rate and reticulocyte counts. Similarly, Maier-Redelsperger et al. [25] have reported lower log(RBC-Hb/Ret-Hb) values, a marker of red cell survival in those with albuminuria. The relationship between albuminuria and reticulocytosis will be confounded by measurement techniques, red cell survival as well as variations in marrow activity. Our findings may suggest lower marrow activity in those with decreasing renal function, possibly mediated by decreasing erythropoietin levels.

White blood cell count is also known to be a marker of severity in SCD and is a strong predictor of mortality [27]. This study has shown it to be associated with increasing albuminuria in both genotypes indicating a possible inflammatory mechanism that is implicated in the pathogenesis of sickle nephropathy. It is well known that part of the pathogenesis of vascular occlusion in SCD is due to increased leukocyte adherence to its endothelium [28]. Asymptomatic bacteriuria has been shown to be higher in SCD [29] with a prevalence of 5% in this study cohort, and its association with subsequent nephropathy remains to be determined; however, the presence of subclinical infections associated with high leukocyte counts may be one mechanism behind sickle nephropathy. The decrease in neutrophils secondary to hydroxyurea therapy may therefore be a beneficial therapy in slowing progression of nephropathy, especially as it has been that those with high leukocyte count tend to show a greater positive response to hydroxyurea therapy [30].

In summary, even though the cross-sectional design of this study does not allow for causal inferences to be drawn, patients with rising blood pressure, higher white blood cell counts and those with HbSS may be more prone to renal dysfunction and an early marker of disease is albuminuria. Prospective studies are needed to establish a causal relationship as regular screening may then help identify renal disease early and timely interventions may help delay the progression of nephropathy.

## References

[1] Barton EN, Sargeant LA, Samuels D, Smith R, James J (2004). A survey of chronic renal failure in Jamaica.. West Indian Med J.

[2] Thomas AN, Pattison C, Serjeant GR (1982). Causes of death in sickle-cell disease in Jamaica.. Br Med J (Clin Res Ed).

[3] Powars DR, Elliott-Mills DD, Chan L, Niland J, Hiti AL (1991). Chronic renal failure in sickle cell disease: risk factors, clinical course, and mortality.. Ann Intern Med.

[4] Saborio P, Scheinman JI (1999). Sickle cell nephropathy.. J Am Soc Nephrol.

[5] Guasch A, Cua M, Mitch WE (1996). Early detection and the course of glomerular injury in patients with sickle cell anemia.. Kidney Int.

[6] Abbate M, Benigni A, Bertani T, Remuzzi G (1999). Nephrotoxicity of increased glomerular protein traffic.. Nephrol Dial Transplant.

[7] Remuzzi G, Bertani T (1998). Pathophysiology of progressive nephropathies.. N Engl J Med.

[8] Sklar AH, Campbell H, Caruana RJ, Lightfoot BO, Gaier JG (1990). A population study of renal function in sickle cell anemia.. Int J Artif Organs.

[9] Wigfall DR, Ware RE, Burchinal MR, Kinney TR, Foreman JW (2000). Prevalence and clinical correlates of glomerulopathy in children with sickle cell disease.. J Pediatr.

[10] Sesso R, Almeida MA, Figueiredo MS, Bordin JO (1998). Renal dysfunction in patients with sickle cell anemia or sickle cell trait.. Braz J Med Biol Res.

[11] Falk RJ, Scheinman J, Phillips G, Orringer E, Johnson A (1992). Prevalence and pathologic features of sickle cell nephropathy and response to inhibition of angiotensin-converting enzyme.. N Engl J Med.

[12] Schmitt F, Martinez F, Brillet G, Giatras I, Choukroun G (1998). Early glomerular dysfunction in patients with sickle cell anemia.. Am J Kidney Dis.

[13] Allon M (1990). Renal abnormalities in sickle cell disease.. Arch Intern Med.

[14] Ataga KI, Orringer EP (2000). Renal abnormalities in sickle cell disease.. Am J Hematol.

[15] Aoki RY, Saad ST (1995). Enalapril reduces the albuminuria of patients with sickle cell disease.. Am J Med.

[16] Foucan L, Bourhis V, Bangou J, Merault L, Etienne-Julan M (1998). A randomized trial of captopril for microalbuminuria in normotensive adults with sickle cell anemia.. Am J Med.

[17] Asnani MR, Fraser RA, Lewis NA, Reid ME (2010). Depression and loneliness in Jamaicans with Sickle Cell Disease.. BMC Psychiatry.

[18] Aoki RY, Saad ST (1990). Microalbuminuria in sickle cell disease.. Braz J Med Biol Res.

[19] Thompson J, Reid M, Hambleton I, Serjeant GR (2007). Albuminuria and renal function in homozygous sickle cell disease: observations from a cohort study.. Arch Intern Med.

[20] Guasch A, Navarrete J, Nass K, Zayas CF (2006). Glomerular involvement in adults with sickle cell hemoglobinopathies: Prevalence and clinical correlates of progressive renal failure.. J Am Soc Nephrol.

[21] Pegelow CH, Colangelo L, Steinberg M, Wright EC, Smith J (1997). Natural history of blood pressure in sickle cell disease: risks for stroke and death associated with relative hypertension in sickle cell anemia.. Am J Med.

[22] Kato GJ, McGowan V, Machado RF, Little JA, Taylor J VI (2006). Lactate dehydrogenase as a biomarker of hemolysis-associated nitric oxide resistance, priapism, leg ulceration, pulmonary hypertension, and death in patients with sickle cell disease.. Blood.

[23] Taylor JG VI, Nolan VG, Mendelsohn L, Kato GJ, Gladwin MT (2008). Chronic hyper-hemolysis in sickle cell anemia: association of vascular complications and mortality with less frequent vasoocclusive pain.. PLoS One.

[24] Gurkan S, Scarponi KJ, Hotchkiss H, Savage B, Drachtman R (2010). Lactate dehydrogenase as a predictor of kidney involvement in patients with sickle cell anemia.. Pediatr Nephrol.

[25] Maier-Redelsperger M, Levy P, Lionnet F, Stankovic K, Haymann JP (2010). Strong association between a new marker of hemolysis and glomerulopathy in sickle cell anemia.. Blood Cells Mol Dis.

[26] Haymann JP, Stankovic K, Levy P, Avellino V, Tharaux PL (2010). Glomerular hyperfiltration in adult sickle cell anemia: a frequent hemolysis associated feature.. Clin J Am Soc Nephrol.

[27] Hagar W, Vichinsky E (2008). Advances in clinical research in sickle cell disease.. Br J Haematol.

[28] Okpala I (2004). The intriguing contribution of white blood cells to sickle cell disease - a red cell disorder.. Blood Rev.

[29] Cumming V, Ali S, Forrester T, Roye-Green K, Reid M (2006). Asymptomatic bacteriuria in sickle cell disease: a cross-sectional study.. BMC Infect Dis.

[30] Charache S (1997). Mechanism of action of hydroxyurea in the management of sickle cell anemia in adults.. Semin Hematol.

